# Supplementary material for: Using machine learning as a surrogate model for agent-based simulations
Source: PLoS One. 2022 Feb 10;17(2):e0263150. doi: 10.1371/journal.pone.0263150 (PMC8830643; doi:10.1371/journal.pone.0263150)
Supplement: Attachment — Submitted filename: PLoS Response to reviewers.docx [file pone.0263150.s001.docx]

Dear Dr Bouffanais,

Thank you for sharing the reviewer comments, which we have found helpful and constructive, and for giving us the opportunity to revise our submission. We have provided a detailed response to the reviewers’ comments below.

***Reviewer 1: Major Points***

***1. The authors claim that machine-learning methods have rarely been employed in the ABM literature (pag. 3). This is partially true, but I suggest them looking at the recent survey by Dahlke et al 2020. In addition, please note that Lamperti et al. 2018, which the authors cite, conduct an exercise which is similar to the one proposed in this paper by comparing surrogate models obtained with GP and Boosted Trees (through XGBoost) across different metrics and using various sample sizes. I suggest the authors to discuss their results in light of those obtained in Lamperti et al. 2018 and in the rest of the literature. I feel this paper consolidates - and substantially expand - some evidence which was already there: this is a value added.***

We thank the reviewer for this comment and for the references, which we have discussed and included in our manuscript.

In our introductory section on the existing approaches using machine learning in ABMs, following Dahlke et al., we have mentioned the two main existing applications, namely: (i) modelling adaptive agents that can learn from experience through reinforcement learning approaches; and (ii) analysing and post-processing the (often large-scale) outcomes of running a given ABM. Our surrogate modelling approach has been discussed in this context.

We have also improved the section titled “Interpretability of machine-learning models” in light of the recent efforts in the explainability of machine learning predictions. We have mentioned that machine learning techniques, if used to mimic ABMs and coupled with tools for interpretations of the predictions, can shed light on the input-output relationships. However, if the input-output link is of interest, it is paramount to analyse the models with explainability techniques for machine learning models (e.g. SHAP), which can quantify the influence that each factor has on the model outcome. For a more in-depth discussion on the advantages and disadvantages of these approaches in the context of ABMs, we have then referred the reader to the recent systematic literature review and discussion by Dahlke et al., as suggested by the reviewer.

Furthermore, when we discuss the results in the revised version of the manuscript, we now refer to Lamperti et al. (2018), who convincingly showed that Kriging was outperformed by machine learning surrogates (XGBoost in particular) in terms of precision and computational efficiency when predicting the surface of the Island growth model. We believe it is important to compare methods also by evaluating the different challenges posed by different types of ABMs. For instance, in our Linked Lives case study, gradient boosted trees perform consistently well, but they are outperformed by neural networks. For these reasons, we preferred to keep the comparison open to several methods showing all the results, which are likely to differ depending on the type and complexity of ABM under investigation. In the revised version, we have highlighted this aspect in the Conclusions section.

***2. One crucial element in the analysis of stochastic ABMs embedding a time component is ergodicity, which I intend here as the presence of a single statistical equilibrium to which various runs (i.e. model runs with different seeds and same parameter configuration) approximately converge after a warm-up period (see for example Grazzini 2012; Vandin et al. 2021). My feeling is that surrogate modeling needs ergodicity as a prerequisite. It seems to me that the model considered by the author is deterministic (apart from the choice of initial conditions); however, several ABMs embed random draws during the simulation, and I feel the authors need to discuss whether their exercise and results can be extended to this class of models and under which conditions.***

This is a very interesting point for discussion. We agree with the general principle that ergodicity would be beneficial for surrogate modelling, as it would reduce the code uncertainty. However, we agree with O’Hagan that in practice, stochastic simulators can still be used in surrogate modelling:

“The distinction [between stochastic and deterministic] is again rather artificial, because even a stochastic simulator could be made deterministic by making the random number seed an input.” (O'Hagan 2006).

This being the case, if during a parameter sweep a modeller discovers that their simulator lacks ergodicity and parameter changes lead to significant differences in the output, then the random seed can be included as an input to the emulator. As with GP emulation, we assume in this analysis that modellers will be dealing with simulators that are deterministic, and therefore produce the same results at identical values of the random seed. As noted by the reviewer, the Linked Lives model is indeed deterministic.

We have added a discussion on this point in a new subsection entitled “Properties of the Linked Lives model”:

“Properties of the Linked Lives model

The Linked Lives model would be classified in the emulation literature as a deterministic simulator, meaning that a given set of parameter values will produce the same results, presuming that the same random number seed is used. Many simulations are of this type, as they will be implemented in standard programming languages which use pseudo-random number generators.

In previous explorations, the Linked Lives model has demonstrated ergodicity, meaning that the simulation tends to converge to similar outputs at identical parameter values, even if the random seed is different. However, we note that this property, while useful in that it suggests less code uncertainty is present in the simulation, is not essential to the use of surrogate modelling techniques. If the random seed has a significant influence on model outputs, surrogates like Gaussian process emulators can still be constructed. In this context, the distinction between stochastic and deterministic models can be sometimes artificial, as a stochastic simulator can be interpreted as deterministic when the random number seed becomes an input (O’Hagan, 2006).

Therefore, when generating runs for a simulation that has a high degree of stochasticity, modellers should record the used random seed with each run to enable the inclusion of those seeds as an additional input when training the surrogate. In this way, the simulation can be treated as a deterministic model.”

***3. Is the interpretability of the ML surrogate valuable? The authors acknowledge that surrogates might be useful not just to improve the efficiency of simulations (which is necessary for - e.g. - global sensitivity analysis), but to help understand the model behavior, which is often complex (pag.4). However, when discussing their results, they do not mention the issue of interpretability, which I feel as relevant instead. For example, decision trees or random forests might help detect which parameters are more relevant to obtain behaviors aligned with the actual model or to models’ fit with the empirical data, therefore helping understand the model itself and its “sensitive” parts. To the eyes of a modeler this is fundamental for calibration and sensitivity analysis. To the contrary, ANNs can difficulty offer such information. I would suggest the authors to build on their observation of pag. 4 and discuss this issue in their Discussion and Conclusions sections.***

Following these suggestions, and also in response to Point 1, we improved our section on interpretability in machine learning surrogates. We agree that this discussion could be further developed in the conclusion of the paper. Therefore, we expanded the final paragraph as follows:

“Before these alternative surrogate modelling methods can become widely adopted, we must find efficient and effective ways of making use of these more accurate surrogate models. Unlike GPs, methods like neural networks, non-linear SVM and gradient-boosted trees do not bring with them insightful uncertainty quantification measures `for free'. In this respect, more effort will need to be spent on the interpretation of the surrogates, which can be challenging when the surrogate models are complex.

However, the often-repeated notion that neural networks or other ML approaches are less interpretable than linear methods is not so clear in practice (Lipton, 2018). Therefore, we avoided classifying these methods by interpretability, as the concept itself is poorly defined and its relative importance will vary enormously depending on the application, whereas runtimes and performance are more broadly useful measures. In addition, as mentioned above, even an opaque surrogate can enable powerful analyses to be performed more quickly, and thus aid in the interpretation of the original simulation.

Interpretable machine learning has become a field in its own right in recent years, and powerful techniques for interpretable ML models are now widely accessible (Du et al., 2019; Molnar, 2019). As specialised libraries for ML-based surrogate modelling become available, interpretability tools can be integrated into the process, enabling modellers to generate additional understanding from their surrogates as well as more rapid sensitivity analyses. In later extensions of this work, we will investigate the use of these tools in ML-based surrogate modelling of agent-based simulations.

Following on from this study, we will focus on developing libraries and examples of how to use ML-based surrogate models to generate deeper insights into the behaviour of complex ABMs. This may significantly enhance future modelling efforts in the ABM community by increasing the accessibility of these techniques. These additional methods for simulation analysis would serve to fill the gaps where GPs are unable to produce usable surrogates, ensuring that detailed sensitivity and uncertainty analysis become the norm in agent-based modelling studies.”

***4. Figure 3a is relevant. It shows that GP emulators (as implemented by the authors) have difficulties. I feel it would be relevant to show (i) the ground truth to which the emulator should be compared and (ii) similar charts for the other surrogates. Also, I would add a note of caution; if the ground truth behavior (i.e. how the actual ABM responds to parameter) is relatively smooth or even linear, learning should be easier. I wonder how the different methods perform when facing highly non-linear model dependence on parameters. I suggest providing – at least - some discussions.***

We would like to thank the reviewer for this remark. This is a very interesting point, which we have discussed in much more detail in the revised version. GPs are limited in their capacity to be a surrogate model of certain kinds of complex simulation models, in that they assume that simulation output varies smoothly in response to changing inputs. In the case of complex ABMs, this assumption often fails as model outputs can change in unexpected ways in response to small variations in input parameter values. Our exemplar ABM falls into this category, meaning that even though other critical GP assumptions still hold (the model is deterministic, and has a relatively low number of inputs), the emulator is unable to fit a GP model to the simulation data. We run a revised PCA analysis to show that, in our case, determining the impact of the input variables on the final output variance is challenging. This may also have contributed to the low performance of this initial GP emulator attempt. These remarks have been included at the beginning of the Results section.

More specifically, to supplement the discussion and provide more interpretation on how the various parameters affect the final output, we also focused more on the results of the PCA, to add additional interpretation to the results. To this end, the factor maps have been replaced with variable contribution maps. The variable, eigenvector and eigenvalue results have been included as supplementary material on the GitHub repository. The following additional text has been added to the Investigation of Simulation Outputs section:

“At 200 samples the highest contributors to principal component 1 are homeAdultHours and ageOfRetirement. Conversely, at 400 samples the highest contributors to principal component 1 are maleAgeCareScaling and workingAdultHours.  HomeAdultHours governs the amount of hours unemployed adults are able to devote to care, workingAdultHours determines how many hours working adults can allocate to care, and ageOfRetirement determines when agents are able to retire from work, thus freeing up more hours for care.  These variables being the highest contributors suggests that increased availability of informal care hours for adults, either by increasing their allocation generally or through retirement, has a substantial impact on unmet care need.  The parameter maleAgeCareScaling quantifies the amount of care need exhibited in the male agent population as they age, so a large change in this value can significantly impact the general level of unmet care need in the agent population.”

***5. This is probably the main concern I have. I have issues at understanding why the accuracy of surrogate models - e.g. GP and Boosted trees - vary so largely when increasing sample size, especially from 800 samples to 1600 samples. The performance seems rather consistent from 200 to 800 samples, and then it drops quite dramatically for 1600 samples. This is rather counter-intuitive to me. What happens, e.g., at 1200 samples? Can they author provide a convincing explanation of this issue, which greatly affects the results proposed by the paper.***

As the reviewer correctly points out, the challenge faced by our approach of approximating ABM output is that multiple runs of the same configuration may give different output results. As a result, there is an intrinsic "noise" that the machine learning methods need to filter out while carrying out the predictions. Given the nature of ABMs, each parameter change could lead to substantial changes in the behaviour of the model, since the interactions of these parameters are complex.

Notwithstanding these challenges, the Gradient Boosted trees are consistently reducing the MSE Loss with the increasing number of samples. After 800 runs, the error starts to increase due to overfitting, which is a common disadvantage of tree-based methods. High sample size is also likely to require more careful optimisation of the hyperparameters, which are harder to tune in XGBoost compared to the other methods. The effect of overfitting is avoided with Random Forest (also tree-based), although the absence of the Boosting approach makes Random Forest a less suitable choice due to the lower performance on the MSE loss. Furthermore, Gradient Boosted Trees are prone to fluctuations due to outliers (common in ABM-generated results), as every tree is built to improve and mitigate the error obtained by the previous trees. As a result, having a high number of ABM-generated samples with a difficult or irregular pattern to extract poses challenges to boosting approaches, undermining their scalability.

While our paper provides an exploratory analysis, we believe it is important to mention that our results highlight that further hyperparameter optimisation and a strategy to reduce overfitting should be implemented after having selected the method. We have now included this discussion in ‘Investigation of the Simulation Outputs’.

***Reviewer 2: Major Points***

***1. Most agent-based models are stochastic, meaning each run will lead to different output values even if input values are the same. The approach in this paper does not capture uncertainty generated by multiple runs of the same configuration.***

As noted above in our response to Reviewer 1’s point 2, we take the view expressed in O'Hagan 2006 on this important issue, namely that the distinction between a deterministic and stochastic simulator can be somewhat artificial. For instance, stochastic simulations can be made deterministic by taking the random seed as an additional input.  We also note that GP emulation explicitly accounts for uncertainty in the output of the original simulation, referred to as code uncertainty.

Regarding the Linked Lives model specifically, we follow GP emulation practices and generate space-filling sets of parameter values for a deterministic simulator using the LP-Tau method. As a result, the outputs should provide a suitably comprehensive picture of the model's behaviour throughout the parameter space.

In future work, we aim to delve deeper into the use of ML techniques other than GP emulation, and investigate how best to account for code uncertainty using these methods. As noted in the conclusion, GP emulation has a great advantage in that uncertainty quantification is a fundamental part of the method; however, as we demonstrate in our initial investigation of the model, GP emulators are not always able to achieve a good fit with complex ABMs. We hope that this paper can suggest which alternative ML methods are most promising for this particular use case.

***2. would recommend finding the best performing ML technique first. Then, I would compare it with GP and other surrogate modeling techniques. Speaking of other techniques, Kriging is also a popular surrogate modeling technique to compare against.***

We thank the reviewer for pointing this out. While this would be an interesting alternative approach, in our paper we attempted to provide a survey of numerous ML methods, discussing their strengths and weaknesses specifically for potential use as surrogates. This approach would therefore preclude pre-selection of best-performing methods, as we argue that the best method might in principle differ depending on the specific ABM under investigation.

As both GP emulation and Kriging are forms of Gaussian process regression, here we opted to focus on GP emulation as the main comparator, because GP emulation is found more often in the ABM literature. We included both these points in the Motivations and Conclusions sections in the revised version. Furthermore, when we discuss the results, we also now refer to Lamperti et al. (2018), who convincingly showed that Kriging was outperformed by machine learning surrogates (XGBoost in particular) in terms of precision and computational efficiency when predicting the surface of the Island growth model.

In our Linked Lives case study, gradient boosted trees perform consistently well, but they are outperformed by neural networks. For these reasons, we preferred to keep the comparison open as the results might differ depending on the type and complexity of ABM under investigation. We believe this is an interesting point for discussion, and we have therefore highlighted this aspect in the Conclusions section of the revised version.

***3. Experiment sample size is quite limited. There is only one ABM used and there is only one output value observed. More ABMs (check Comses) and multi outputs can generate interesting and more comprehensive results.***

The model used here was written by one of the authors and has been studied extensively.  Consequently, we were able to provide insightful details and commentary on this model and rewrite the code to more efficiently generate the results we needed.  If we had adopted models used by others, we would have not been able to investigate them at this level of detail, and we would have introduced substantially more practical difficulties in understanding the code and making the needed modifications to output generation and recording. We have provided additional rationale for this choice in response to Minor Point 5 below.

We believe that the use of one output was to facilitate comparison with GP emulation approaches, which typically focus on a single main output as the method becomes significantly more complex when multiple outputs of interest are investigated.  As stated in the paper, we aim to demonstrate that emulation can be used effectively and quickly to gain insight into complex ABMs, even when time, expertise and resources are limited; this statement becomes progressively less true as the complexity of the emulation process increases.  We would welcome follow-up work targeted at simulation specialists that investigated more sophisticated emulation approaches. We hope that our paper can provide a useful investigation of the most promising methods for further investigation.

***Reviewer 2: Minor Points***

***“ABM-based modelling” redundant words.***

Thanks, “ABM-based modelling” has been now changed into “agent-based modelling”.

***1. I believe the term “emulate” is not the right one to use in this context. Emulate indicates being “in place of” something and replacing it. In the context mentioned here there is “approximation” not replacement of the real simulation model. Even though the Gaussian process paper uses the term emulator, it is not the right use here. Surrogate model sounds like a better option.***

We thank the reviewer for bringing this to our attention and have modified the text to use surrogate instead of emulate in all cases except the use of Gaussian Process emulation. In the GP emulator literature, an 'emulator' is defined as 'a statistical approximation of the simulator' (O'Hagan 2006). Here we position GP emulators as not model replacements as such, but statistical approximations for use in sensitivity analysis, and we believe it is more consistent with the existing GP emulation literature to use 'emulator' in this case.

***2. The authors define ABM as both “Agent-Based Modelling” and “Agent-Based Model.” These two are not interchangeable. Please select one and use it consistently.***

The text has been updated so that ABM is only used as an acronym for Agent-Based Model.

***3. The first paragraph in the second page of the article starts describing agent-based modeling without any references. Some introductory references are needed.***

We agree with the reviewer that the introduction could have provided more background knowledge for the reader. We have now added a paragraph early in the Introduction, which provides the reader with a definition of agent-based modelling:

“Agent-based modelling is a computational modelling approach most often applied to the study of complex adaptive systems (De Marchi and Page, 2014). ABMs typically represent individuals directly, and situate these agents in a virtual environment of some kind. These agents then engage in varied behaviours encoded in a set of decision rules that drive their actions in response to behavioural, environmental and social change. The resultant complex interactions between agents and their environments can lead to emergent behaviours, in which the patterns of behaviour seen at the population level have new properties that are not straightforwardly attributable to individual-level actions (Janssen and Ostrom, 2006).“

***4. “When ABMs are highly complex, performing these kinds of analyses becomes both time- and cost-prohibitive, potentially leading some modellers to truncate these analyses or eliminate them entirely.” I would add the implications of this statement which is related to verification and validation.***

We fully agree that this behaviour has implications for verification and validation, which are already difficult tasks in an ABM context. We explored this issue in more detail in a new paragraph we included in the Introduction:

“When such analyses are not performed, model verification and validation can become more challenging. Sensitivity analyses provide insight into the contributions made by model parameters, which can be valuable for verifying whether the model processes driven by those parameters are functioning correctly. ABMs simulate multi-level complex systems, which may be multi-realizable, meaning that the macro-level phenomena of interest may be generated in multiple ways at the individual level (Epstein 2006, Sawyer 2005). That being the case, validation for ABMs can be particularly challenging, as the ABM may be only one of many possible explanations for the macro-level phenomena. Sensitivity analysis can be used to provide a more complete description of the model's behaviour, which can help the modeller to justify why their implementation constitutes a useful explanation of the underlying system. Without such analyses, validation relies on face validity and matching input-output transformations to real data, which cannot provide evidence on whether the underlying model processes and assumptions are valid.”

***5. The paper calls the case study as moderate-complexity ABM. It’s not clear how model complexity is measured. Are there any objective techniques to do so?***

We are not aware of a relevant measure of model complexity that could be used to quantify this statement. We used the term 'moderate complexity' to indicate that the model is not a 'toy model', nor is it a very large model with hundreds of parameters; runtimes are on the order of minutes rather than seconds or hours/days. We felt this to be more useful than a model positioned at either extreme, as toy models are typically targeted at theoretical questions and are easier to analyse using traditional methods, while very large models are often avoided in an ABM context, given the focus on explanatory aims in many ABM studies. To clarify this point, we have added a paragraph to the section entitled The ‘Linked Lives’ Agent-Based Model’, which explains this terminology in greater detail:

“To present a useful comparison of methods for generating surrogate ABMs, we chose to use an exemplar ABM which is neither a simplistic `toy model', nor a highly-detailed simulation with hundreds of parameters. Simplistic ABMs may generate behaviour that is easier to replicate, meaning they would not present a sufficient test of the capabilities of the emulators, and in practice some simplistic models could be analysed relatively effectively using traditional methods, given the short runtimes. Conversely, high-complexity ABMs may provide a very robust test of the capabilities of ML-based emulation. However, a highly complex model would have taken impractical amounts of CPU time to generate output data and would not be representative of the typical empirical ABM, where overly complex models are often avoided to enable the model to provide explanatory insight into the system of interest. Therefore, for reasons of applicability, practicality, and replicability we chose to use a model between these two extremes, which we call `moderately complex', with runtimes on the order of several minutes and ten configurable parameters.”

***6. Why spider plot :) Barplot would work better.***

We chose to use spider plots as they enabled us to quickly make simultaneous comparisons of the emulation methods on both speed and accuracy. This is also considering that the actual numerical value is already reported in the table within Figure 1. Given that the goal of emulation is to significantly reduce CPU time requirements relative to repeated runs of the original simulation, we report both the accuracy of the approximations and the runtimes required to generate them. The spider plot provides a straightforward visual comparison of both measures for each method in each scenario. An advantage of the spider plot compared to the bar plot in our case is that it allowed us to quickly detect outliers, as they affect the shape of the polygon.

***7. In figure 5, is the dotted line y=x? If so, please mention.***

Thanks, the caption in Figure 5 has been updated to include that the dotted line is *y=x*.
